# Identification, functional characterization, and pharmacological profile of a serotonin type-2b receptor in the medically important insect, *Rhodnius prolixus*

**DOI:** 10.3389/fnins.2015.00175

**Published:** 2015-05-19

**Authors:** Jean-Paul V. Paluzzi, Garima Bhatt, Chang-Hui J. Wang, Meet Zandawala, Angela B. Lange, Ian Orchard

**Affiliations:** ^1^Department of Biology, York UniversityToronto, ON, Canada; ^2^Department of Biology, University of Toronto MississaugaMississauga, ON, Canada

**Keywords:** serotonin, 5-hydroxytryptamine (5-HT), G protein-coupled receptor (GPCR), diuresis, dorsal vessel, Malpighian tubules, serotonergic signaling, cardioacceleratory activity

## Abstract

In the Chagas disease vector, *Rhodnius prolixus*, two diuretic hormones act synergistically to dramatically increase fluid secretion by the Malpighian tubules (MTs) during the rapid diuresis that is initiated upon engorgement of vertebrate blood. One of these diuretic hormones is the biogenic amine, serotonin (5-hydroxytryptamine, 5-HT), which controls a variety of additional activities including cuticle plasticization, salivary gland secretion, anterior midgut absorption, cardioacceleratory activity, and myotropic activities on a number of visceral tissues. To better understand the regulatory mechanisms linked to these various physiological actions of serotonin, we have isolated and characterized a serotonin type 2b receptor in *R. prolixus*, Rhopr5HTR2b, which shares sequence similarity to the vertebrate serotonin type 2 receptors. *Rhopr5HTR2b* transcript is enriched in well-recognized physiological targets of serotonin, including the MTs, salivary glands and dorsal vessel (i.e., insect heart). Notably, *Rhopr5HTR2b* was not enriched in the anterior midgut where serotonin stimulates absorption and elicits myotropic control. Using a heterologous functional receptor assay, we examined Rhopr5HTR2b activation characteristics and its sensitivity to potential agonists, antagonists, and other biogenic amines. Rhopr5HTR2b is dose-dependently activated by serotonin with an EC_50_ in the nanomolar range. Rhopr5HTR2b is sensitive to alpha-methyl serotonin and is inhibited by a variety of serotonin receptor antagonists, including propranolol, spiperone, ketanserin, mianserin, and cyproheptadine. In contrast, the cardioacceleratory activity of serotonin revealed a unique pharmacological profile, with no significant response induced by alpha-methyl serotonin and insensitivity to ketanserin and mianserin. This distinct agonist/antagonist profile indicates that a separate serotonin receptor type may mediate cardiomodulatory effects controlled by serotonin in *R. prolixus*.

## Introduction

The widespread presence and diverse biological roles of the indoleamine, serotonin (5-hydroxytryptamine, 5-HT), spans protozoans, plants and the vast majority of metazoans, thus attesting to its evolutionary significance (see Turlejski, 1996). In mammals, and more specifically in humans, serotonin acts on many organ systems via 7 families of serotonin receptors (5-HTR_1−7_) that constitute numerous receptor isoforms (see Pytliak et al., 2011; Verlinden et al., 2015). The serotonin receptor, 5-HTR_3_, is a ligand-gated channel whereas all other serotonin receptor classes have been characterized as G-protein coupled receptors (GPCRs) (Millan et al., 2008). Released as both a neurotransmitter and a hormone, the functions of serotonin are extensive, ranging from regulating the mechanics of behaviors like learning, memory, perception, fear and appetite, to mediating a plethora of physiological processes (see Berger et al., 2009; Verlinden et al., 2015).

The similarity in serotonin's regulatory functions in vertebrates as well as invertebrates is noteworthy. To name a few, serotonin is associated with learning and memory in sea slugs (Rahn et al., 2013), social and anxiety-like behavior in crayfish (Momohara et al., 2013; Fossat et al., 2014) and aversive behavior in the nematode, *Caenorhabditis elegans* (Curran and Chalasani, 2012). Serotonin is also a key modulator of feeding-related behaviors, including salivation, bite-like movements, pharyngeal peristalsis, and control of blood meal ingestion in a blood-feeding aquatic invertebrate, the medicinal leech, *Hirudo medicinalis* (Lent and Dickinson, 1987, 1988; Lent et al., 1988).

In insects, serotonin influences feeding-associated behaviors in various species including the locust (Ali et al., 1993; Molaei and Lange, 2003), blowfly (Baumann and Walz, 2012), honeybee (French et al., 2014), cockroach (Troppmann et al., 2007), as well as an ant (Falibene et al., 2012). Studies in *Drosophila melanogaster* and *Apis mellifera* have linked serotonin to development and various behaviors associated with central pattern generators, such as olfaction, learning, memory and circadian rhythms (Blenau and Thamm, 2011; Johnson et al., 2011). The immunohistochemical mapping of serotonergic neurons in the nervous systems of several dipteran, orthopteran, lepidopteran, blattarian, and hemipteran species further emphasizes serotonin's role as a neurotransmitter and neurohormone in insects (Nassel, 1988; Bicker, 1999; Miggiani et al., 1999; Homberg, 2002; Siju et al., 2008).

*Rhodnius prolixus*, a chief vector of Chagas disease, depends on its serotonergic system to successfully complete blood meal engorgement and, even more importantly, to regulate haemolymph osmolarity after its dramatic feeding bout. On average, a fifth instar *R. prolixus* can consume blood meals that are 10 times its unfed body weight and then promptly eliminates excess water and ions via rapid hormone-controlled diuresis (Orchard, 2006, 2009). Serotonin is a principal diuretic hormone in *R. prolixus* and haemolymph titres of serotonin rise dramatically from low nanomolar (<10 nM) to high nanomolar (>100 nM) levels within 5 min of feeding (Lange et al., 1989; Maddrell et al., 1991). Upon its release via serotonergic neurohaemal sites, serotonin stimulates diuresis, muscle contractions of the dorsal vessel, salivary glands, esophagus (foregut), anterior midgut (i.e., crop), and hindgut (Orchard and Te Brugge, 2002; Orchard, 2006). In addition, serotonin is involved with the plasticization of the cuticle and the expulsion of waste (Orchard et al., 1988; Lange et al., 1989).

Along with a corticotropin-releasing factor (CRF)-like peptide, RhoprCRF/DH, serotonin is involved in the production of primary urine by stimulating secretion of excess water and ions by the Malpighian tubules (MTs) (see Martini et al., 2007). Unlike most other tissues where serotonin is known to play a role, the MTs lack innervation and are thus influenced by the rise in the haemolymph levels of serotonin, acting as a neurohormone (Lange et al., 1989). The presence of serotonin receptors on the epithelial cells of the MTs is therefore critical to the normal course of rapid post-feeding diuresis in *R. prolixus*. Interestingly, serotonin and RhoprCRF/DH have been shown to synergistically increase fluid secretion in the MTs, and both appear to be at least partially dependent on both intracellular calcium and cAMP-mediated signal transduction pathways (Paluzzi et al., 2013). A recent study corroborating the requirement of both intracellular calcium and cAMP revealed that serotonin triggers calcium signaling (derived specifically from intracellular stores), which is mediated by cAMP and cAMP-dependent kinase, protein kinase A (Gioino et al., 2014).

Based on similarity to the mammalian serotonin receptor families, research in insects has identified six serotonin receptor subtypes, including type 1a, type 1b, type 2a, type 2b, type 7, (Tierney, 2001; Vleugels et al., 2013, 2014; Verlinden et al., 2015) and a novel type 8, recently identified in the small white butterfly, *Pieris rapae* (Qi et al., 2014). In comparison to the vertebrate receptors, the insect serotonin receptors have different pharmacology and may utilize varying modes of signal transduction (Vleugels et al., 2014; Verlinden et al., 2015). Serotonin receptors have been predicted and/or cloned from several insects and their endogeneous roles are now being explored (Von Nickisch-Rosenegk et al., 1996; Pietrantonio et al., 2001; Dacks et al., 2006b; Hauser et al., 2006, 2008; Troppmann et al., 2010; Gasque et al., 2013; Thamm et al., 2013; Vleugels et al., 2013, 2014).

In spite of the wealth of knowledge on serotonin's physiological roles in *R. prolixus*, surprisingly little is known concerning their endogenous serotonin receptors. In addition, we were interested to see if there might be more than one serotonin receptor in the periphery mediating the physiological effects of serotonin. We chose a tissue that is not directly involved in the rapid post-feeding diuresis. Thus, we examined the effects of serotonin agonists and antagonists on serotonin-stimulated increases in heart beat frequency (see Orchard, 2006). The susceptibility of these serotonin receptors to agonists and antagonists could then be compared to the pharmacology of the cloned receptor. The overall aim of this study was to address this knowledge gap and to begin to identify the serotonin receptors that mediate the various functional activities of serotonin in *R. prolixus*.

## Materials and methods

### Animals

Fifth instar *R. prolixus* were obtained from an established colony at the University of Toronto Mississauga. Insects were reared in incubators at 25°C under high humidity (~50%). Each post-embryonic developmental stage was blood fed through an artificial feeding membrane as described previously (Paluzzi et al., 2015) using defibrinated rabbit blood purchased from a local supplier (Cedarlane Laboratories Inc., Burlington, ON). During dissection of animals to retrieve RNA from different tissues, the insects were bathed in nuclease-free phosphate-buffered saline (PBS) (Sigma-Aldrich, Oakville, ON, Canada) and excised tissues were transferred directly into chilled RNA lysis buffer (see below).

### Isolation of a *R. prolixus* putative serotonin receptor cDNA

Based on the pharmacological sensitivity of serotonin-stimulated fluid secretion by isolated *R. prolixus* MTs to ketanserin (Maddrell et al., 1991; Te Brugge et al., 2001), a selective antagonist of serotonin type-2 receptors (Hedner and Persson, 1988), the *D. melanogaster* type-2A serotonin receptor (Colas et al., 1995) protein sequence (Genbank accession# CAA57429) was used in a local tblastn search of the *R. prolixus* preliminary genome using Geneious 6.1 software (Biomatters Ltd. Auckland, New Zealand) and genomic regions with high scoring matches were used to design several pairs of gene-specific primers (Sigma Aldrich, Oakville, ON, Canada). Using a previously prepared *R. prolixus* Malpighian tubule cDNA library (Paluzzi et al., 2010) as template, only one of these primer pairs (5htR2-forA and 5htR2-revA) was successful (see Table 1; unsuccessful primer pairs are not shown), which amplified an initial 263 bp cDNA product. PCR reactions used ThermoPol Taq Polymerase (New England Biolabs, Whitby, ON, Canada) following manufacturer recommendations and carried out on a PCR System 9700 thermal cycler (Perkin Elmer Applied Biosystems, Carlsbad, CA, USA) using the following settings: initial denaturation for 5 min at 95°C; 35 cycles of 1) denaturation for 1 min at 94°C, 2) annealing for 30 s at 58°C, and 3) extension for 30 s at 72°C; final extension for 10 min at 72°C. The amplified cDNA sequence was cloned into a sequencing vector, pGEM T-Easy (Promega Corporation, Madison, WI, USA) and bases identified by Sanger sequencing (Center for Applied Genomics, Hospital for Sick Children, Toronto, ON). The putative translation of this preliminary product was compared to the UniProt protein database using the ExPASy Bioinformatics Resource Portal (http://web.expasy.org/blast/) and found to be highly similar to several uncharacterized protein sequences including a *D. melanogaster* orphan receptor (Genbank accession#: AGP51353.1) having similarity to previously annotated serotonin receptors (Hauser et al., 2006) and recently classified as a serotonin receptor type-2B (Gasque et al., 2013). The *D. melanogaster* 5HTR-2B sequence was used for *in silico* screening of the *R. prolixus* genome (as described above) that yielded several high scoring matches. Similar to the *in silico* screen using the type 2A sequence as bait, many of the high-scoring candidate matches to the serotonin receptor type-2B localized to a single supercontig (GL563092) and so gene-specific primers (Sigma Aldrich, Oakville, ON, Canada) were designed to isolate a larger portion of this candidate *R. prolixus* 5HTR2 cDNA sequence (see Table 1) following PCR conditions similar to those described above using the ThermoPol Taq Polymerase (New England Biolabs, Whitby, ON, Canada). These efforts extended the partial cDNA sequence to a size of 2003 bp but which nonetheless remained incomplete on the 5′ and 3′ ends lacking start and stop codons, respectively.

**Table 1:** **Primer sequences used in this study**.

**Oligo name**	**Oligo sequence (5′-3′)**	**Description (cDNA nucleotide binding site)**
5htR2-forA	TCGCGCACTTCATCTCG	Cloning initial partial cDNA (1969-1985)
5htR2-revA	TTCTTGAATGCTTGTCGAAAC	Cloning initial partial cDNA (2231-2211)
5htR2-for1	GCAGCTGGCAACATCC	Further cloning partial cDNA (250-265)
5htR2-for2	AGTTTACTGTTTGGCGTGG	Further cloning partial cDNA (417-435)
5htR2-for3	CAAATACTATAGTAACATGGGATTCC	Further cloning partial cDNA (1688-1713)
5htR2-rev1	CGATATCGACACAATAAGACTTTC	Further cloning partial cDNA (2252-2229)
5htR2-rev2	CTAAGTGGAAGACTCATAGCTATCG	Further cloning partial cDNA (614-590)
5htR2-rev3	GCATGACGAGTATGGCGAC	Further cloning partial cDNA (370-352)
5htR2_3raceF1	GAAGGAAAGCCAATGAAGAAGATAGG	3′ RACE PCR (1211-1236)
5htR2_3raceF2	GTATTCGATTTTGTCACATGGTTAGG	3′ RACE PCR (2134-2159)
5htR2_3raceF3	TAAAGTGTTTCGACAAGCATTCAAG	3′ RACE PCR (2205-2229)
5htR2_3raceR	TCTTGAATGCTTGTCGAAACAC	3′ RACE PCR positive control (2230-2209)
5htR2_5raceR1	TGCACCAAACTGTTGTAAAGC	5′ RACE PCR (1047-1027)
5htR2_5raceR2	CGCTCGCCCAACC	5′ RACE PCR (835-823)
5htR2_5raceR3	GCATGACGAGTATGGCGAC	5′ RACE PCR (370-352)
5htR2_5raceF	GCAGCTGGCAACATCC	5′ RACE PCR positive control (250-265)
5htR2-ORF-F1	GGCCAAGAAGAAGAGGATGTG	Amplification of complete open reading frame (153-173)
5htR2-ORF-R1	GTTATTGTTACATCTGCCTACGTTC	Amplification of complete open reading frame (2297-2273)
5htR2-ORF-kozak	GCCACCATGTGCAGTGATACAACAGG	Addition of Kozak translation initiation sequence into expression construct (169-188)
5htR2-ORF-R2	CTACGTTCTAGGTGTCCAGCG	Expression construct amplification (2280-2260)
5htR2-qPCR-F	GAACAACGGCAGAACTTGG	Sense primer for qPCR located on exon 4
5htR2-qPCR-R	AATGCCCTCCTCTTTGTATGG	Anti-sense primer for qPCR located on exon 5

*Initial oligonucleotide sequences designed based on high-scoring hits from tBLASTn search of preliminary R. prolixus genome database using D. melanogaster serotonin receptor type-2 sequences as query. Oligonucleotide sequences used in screening of cDNA plasmid library and RACE PCR based on partial initial sequence obtained. Final primer sets for verification of complete open-reading frame (ORF) and mammalian expression contruct based on complete cDNA sequenced de novo and sequence comparisons with the preliminary R. prolixus genome assembly*.

In order to elucidate the entire open reading frame of the receptor, we screened a *R. prolixus* MT cDNA library implementing a PCR-based approach including *R. prolixus* 5HTR2b gene-specific primers (based on the incomplete 2003 bp partial cDNA sequence obtained above) and cDNA library plasmid-specific primers described previously (Paluzzi et al., 2010). In addition, to ensure we obtained the most complete cDNA encompassing both the coding sequence (i.e., open-reading frame) and the untranslated regions, we used a Rapid Amplification of cDNA Ends (RACE) approach following manufacturer suggestions (Roche Applied Science, Laval, QC, Canada) as described previously (Paluzzi et al., 2010). For 5′ and 3′ RACE, fresh cDNA was synthesized from 1 μg of total RNA isolated from a pool of fifth instar tissue samples including nervous, gastrointestinal tract and reproductive tissues. Finally, the complete open reading frame (ORF) sequence was amplified using Q5 high-fidelity DNA Polymerase (New England Biolabs, Whitby, ON) from freshly prepared fifth instar *R. prolixus* MT cDNA synthesized from 500 ng total RNA using iScript RT Supermix (Bio-Rad, Mississauga, ON) following manufacturer recommended conditions. Primers used to amplify the complete ORF are listed in Table 1 and high-fidelity PCR cycling conditions were as follows: 98°C for 30 s for initial denaturation, 98°C for 8 s (denaturation), 65.5°C for 20 s (annealing) and 72°C for 60 s (extension) repeated for 35 cycles that was followed by a final extension at 72°C for 2 min. An aliquot of the PCR reaction was visualized using standard agarose-gel electrophoresis and the remaining PCR reactions were column-purified using the EZ-10 Spin Column PCR Products Purification Kit (Bio Basic, Markham, ON). Amplicons were A-tailed to facilitate T/A cloning into pGEM-T Easy (Promega, Madison, WI) sequencing vector as previously described (Paluzzi et al., 2015). Several independent clones were sequenced to ascertain base accuracy (Center for Applied Genomics, Hospital for Sick Children, Toronto, ON). Preparation of expression constructs in pcDNA3.1 mammalian expression vector was carried out similarly to that described previously (Paluzzi et al., 2010), using primers (see Table 1) incorporating the consensus Kozak translation initiation sequence (Kozak, 1986).

### Receptor functional assay to determine ligand specificity

The pcDNA3.1 Rhopr5HTR2b receptor construct was used for transient expression in a recombinant Chinese hamster ovary cell line (CHOK1-aeq) stably expressing the calcium-sensitive bioluminescent protein aequorin (Paluzzi et al., 2012). Controls using vector without the 5HTR2b insert were included to ensure the functional responses observed were specific to the transiently expressed *R. prolixus* 5HTR2b receptor. Approximately 24 h prior to transfection, CHOK1-aeq cells were seeded at high density into T-75 flasks and were subsequently transfected at either 90% confluence using XtremeGENE HP DNA transfection reagent (Roche Diagnostics, Laval, QC) or 70–80% confluence using Lipofectamine LTX (Life Technologies, Carlsbad, CA) following manufacturer recommendations. Approximately 36–48 h after transfection, cells were prepared for the luminescence assay as previously described (Paluzzi et al., 2010). Kinetic luminescent measurements were made using a VICTOR X Light luminescence plate reader (PerkinElmer, Woodbridge, ON) or a Synergy 2 multi-mode microplate reader (BioTek, Winooski, VT).

Putative ligands and other chemicals were purchased from local suppliers (Sigma-Aldrich, Oakville, ON) and included selected biogenic amines (serotonin, tyramine, octopamine, dopamine), agonists (α-methyl serotonin), and antagonists (ketanserin, mianserin, spiperone, cyproheptadine, gramine, propranolol), that were tested either in isolation or in combination as required for each particular experiment. Biogenic amines and agonists were made up in double distilled water at a stock concentration of 100 mM while antagonists were made up in ethanol at a stock concentration of 1 mM.

### Sequence analysis of the Rhopr5HTR-2b receptor

The deduced protein sequence of the *R. prolixus* serotonin type-2b receptor was analyzed for predicted hydrophobic transmembrane domains using the HMMTOP transmembrane topology prediction server [http://www.enzim.hu/hmmtop/; (Tusnady and Simon, 2001)]. In addition, the deduced Rhopr5HTR-2b protein sequence was evaluated for potential phosphorylation sites using the kinase-specific phosphorylation prediction server, NetPhosK 1.0 (Blom et al., 2004).

The *R. prolixus* serotonin type-2b receptor identified here along with selected structurally related orthologous sequences previously identified in other organisms or predicted in genomic databases were used in a comparative investigation of their primary sequences using ClustalW in MEGA 6.0 (Tamura et al., 2013). To determine the relationship among the various serotonin receptors, including the sequence identified in the current study, phylogenetic analyses were performed using the neighbor-joining method (Saitou and Nei, 1987) and maximum-likelihood method based on the Jones-Thornton-Taylor matrix-based model (Jones et al., 1992), which produced trees that had nearly identical topologies. In order to verify the significance in the determined relationships between the serotonin receptor sequences examined in this analysis, we performed a bootstrap test with 1000 iterations (Felsenstein, 1985).

### Rhopr5HTR-2b tissue expression analysis assessed by quantitative PCR (qPCR)

Since we routinely use fifth instar *R. prolixus* as a model to study neuroendocrine-regulated physiological processes related to blood meal engorgement, tissues were dissected from this developmental stage under nuclease-free phosphate buffered saline prepared as previously described (Paluzzi et al., 2008). Specifically, total RNA was purified from tissues dissected from approximately an equal number of male and female fifth-instar *R. prolixus* fed between 4 and 5 weeks previously as fourth-instar nymphs. cDNA was synthesized for quantitative PCR (qPCR) using 500 ng total RNA isolated from a number of different pools of tissues as described previously (Paluzzi et al., 2015) and were subsequently diluted five-fold with nuclease-free water prior to their use as template. For time-course expression profiling related to blood feeding, MTs were isolated from four insects (4 whole tubules per insect totaling 16 tubules) and stored in RNAlater for subsequent RNA extraction. Time points included 0–30, 30–60 min, 2, 4, 6, and 24 h post-blood feeding (via artificial feeding strategy as described above) as well as unfed controls. Total RNA was isolated from each tissue sample using the Pure Link RNA extraction kit (Life Technologies, Carlsbad, CA) and cDNA was prepared as described previously (Paluzzi et al., 2015) using 250 ng total RNA as template. Since optimal primers over exon boundaries were not possible, primers were instead designed over different exons to facilitate amplification of only a 388 bp cDNA product. Specifically, we used a Rhopr5HTR2b sense primer that was located on the fourth exon while the anti-sense qPCR primer was located on the fifth exon (see Table 1 for details), which span a genomic region of over 3.5 kb. SsoFast EvaGreen Supermix with low ROX (Bio-Rad, Mississauga, ON) was used in all qPCR experiments following recommended cycling conditions on an Mx4000 Quantitative PCR System (Stratagene, La Jolla, CA). *Rhopr5HTR-2b* transcript abundance was normalized to the geometric average expression of three reference genes, namely rp49, β-actin and α-tubulin, which were previously validated for transcript expression profiling in various tissues of fifth instar *R. prolixus* (Paluzzi and O'Donnell, 2012). Experiments were repeated in at least three biological replicates that each included no-template and no reverse transcriptase negative controls.

### Dorsal vessel contraction assay

Serotonin's effect on fifth instar *R. prolixus* dorsal vessel contractions was examined through an *ex vivo* bioassay described previously (Sarkar et al., 2003). Briefly, the insects were gently immobilized on soft dental wax dorsal-side down and the cuticle in the ventral abdominal region and gastrointestinal tract were removed to expose the dorsal vessel lying on the inner dorsal surface of the animal. The semi-isolated dorsal vessel preparations were placed into a Sylgard-lined glass petri dish with the inner dorsal surface facing upwards and were secured through the lateral margins of the dorsal cuticle using minuten pins. Electrodes were connected to an impedance converter (UFI model 2991, Morro Bay, CA, USA) and positioned on each side of the dorsal vessel just anterior to the alary muscles associated with the fifth and sixth abdominal segments. The preparations were maintained in 100 μL of physiological saline (pH 7.0; 150 mM NaCl, 8.6 mM KCl, 2 mM CaCl2, 4 mM NaHCO3, 34 mM glucose, 8.5 mM MgCl2, 5 mM HEPES [pH 7.2]) and different test compounds were applied by first removing 50 μL of the normal saline and replacing it with 50 μL of saline containing a two-fold concentration of the compound being tested. Each preparation served as its own control through monitoring dorsal vessel activity during an initial incubation with saline compared to a second treatment with a given test compound.

## Results

### Cloning and gene structure of a *R. prolixus* 5HTR2b receptor

**Figure 1:**
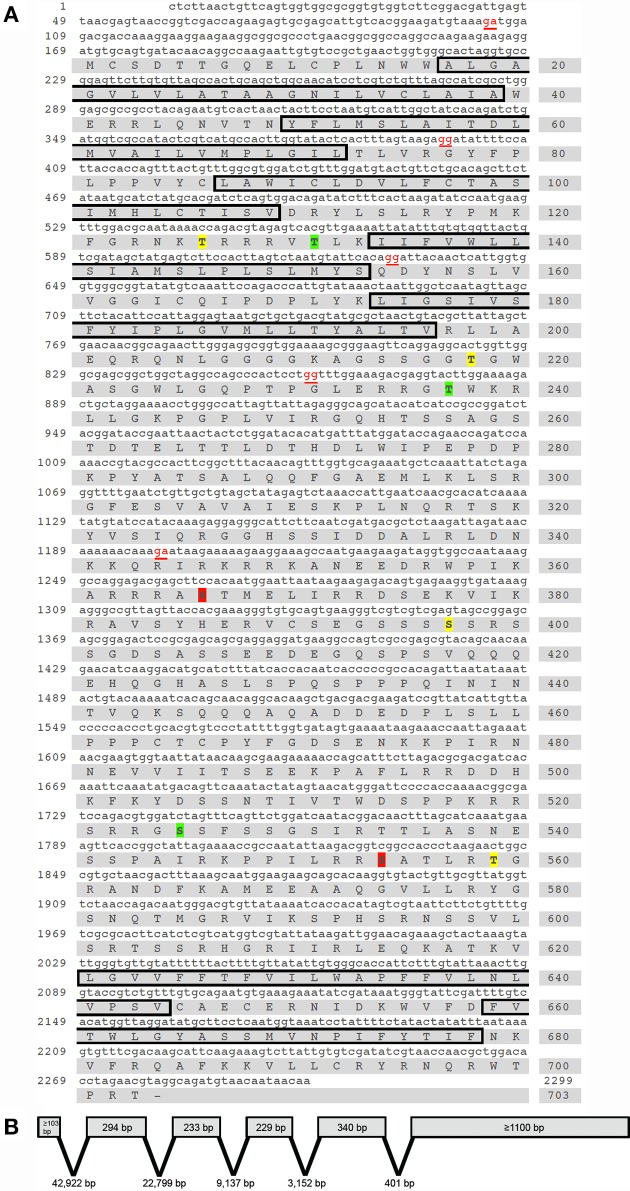
***Rhodnius prolixus* cDNA, deduced protein sequence and predicted gene structure of the serotonin type 2b receptor, Rhopr5HTR2b. (A)** Nucleotide and amino acid numbers are denoted on the left and right side of the sequences, respectively. Exon boundaries are denoted by nucleotides in red text. The predicted hydrophobic alpha-helices that form the seven transmembrane domains are outlined by black boxes. Residues predicted as phosphorylation sites exclusively by protein kinase C are highlighted in yellow (Thr_126_, Thr_218_, Ser_397_ and Ser_559_) while exclusive sites for protein kinase A are highlighted in red (Ser_366_, and Ser_554_) and shared sites are highlighted in green (Thr_131_, Thr_237_ and Ser_525_). **(B)** The Rhopr5HTR2b cDNA identified is produced by six exons and five introns spanning a genomic region of over 80.7 kb.

Using a combined approach involving homology-based *in silico* screening of the preliminary *R. prolixus* genome data and PCR-based strategy to confirm and obtain further sequence information over the 5′ and 3′ regions using RACE PCR, we have identified a *R. prolixus* 5HTR2b (Rhopr5HTR2b) cDNA (Genbank accession KP325472). The total length of the Rhopr5HTR2b cDNA amplified is 2299 bp (Figure 1A), with no additional splice variants identified. Using Geneious 6.1.2, the cloned 2299 bp *R. prolixus* 5HT2b cDNA was used for a local nucleotide BLAST search of the preliminary *R. prolixus* genome dataset to enable detection of exon-intron sites. The gene spans over 80.7 kb and includes six exons with lengths of ≥103 bp; 294, 233, 229, 340 bp and ≥1100 bp as well as five introns with lengths of 42,922, 22,799, 9137, 3152, and 401 bp (Figure 1B). The single open reading frame of 2109 bp produces a protein of 703 amino acid residues (Figure 1A) with an expected molecular mass of 78.7 kDa. In common with other members of the GPCR superfamily, the deduced protein sequence contains seven hydrophobic transmembrane domains. The amino-terminus (N-terminus) has a length of 16 amino acids while the carboxyl terminus (C-terminus) length is 25 amino acids. The open reading frame begins on the second exon, which also yields the first and second transmembrane domains. The third and fourth transmembrane domains are localized to the third exon while the fifth hydrophobic domain is found on the fourth exon. The fifth exon lacks any predicted transmembrane domains but yields the N-terminal region of the third intracellular loop while transmembrane domains six and seven are localized on the sixth and final exon.

### Protein sequence analysis and phylogenetics

Kinase specific phosphorylation sites were predicted using NetPhosK 1.0 (Blom et al., 2004) under a more stringent threshold cut-off (=0.75). A number of phosphorylation sites were predicted based on the deduced protein sequence (Figure 1A), although any functional significance awaits further study. Protein kinase A predicted phosphorylation sites include Thr_131_, Thr_237_, Ser_366_, Ser_525_, and Ser_554_. Predicted protein kinase C phosphorylation sites in the Rhopr5HT2b sequence include Thr_126_, Thr_131_, Thr_218_, Thr_237_, Ser_397_, Ser_525_, and Ser_559_. Within the third hydrophobic transmembrane region, a highly conserved aspartate residue (Asp_93_) is present and is commonly found in aminergic neurotransmitter GPCRs as it is necessary for interaction with the positively charged amine moiety of aminergic ligands permitting receptor-ligand binding (Kristiansen and Dahl, 1996; Kristiansen et al., 2000). Additionally, vertebrate 5HTR type-2 receptors contain a highly conserved serine residue that is localized in close proximity to Asp_93_ (approximately one helical turn), which is a critical residue for interaction with the charged amine side chain of serotonin (Almaula et al., 1996). Notably, this serine residue facilitating receptor-ligand interaction is absent in Rhopr5HTR2b as well as orthologs from other insects.

**Figure 2:**
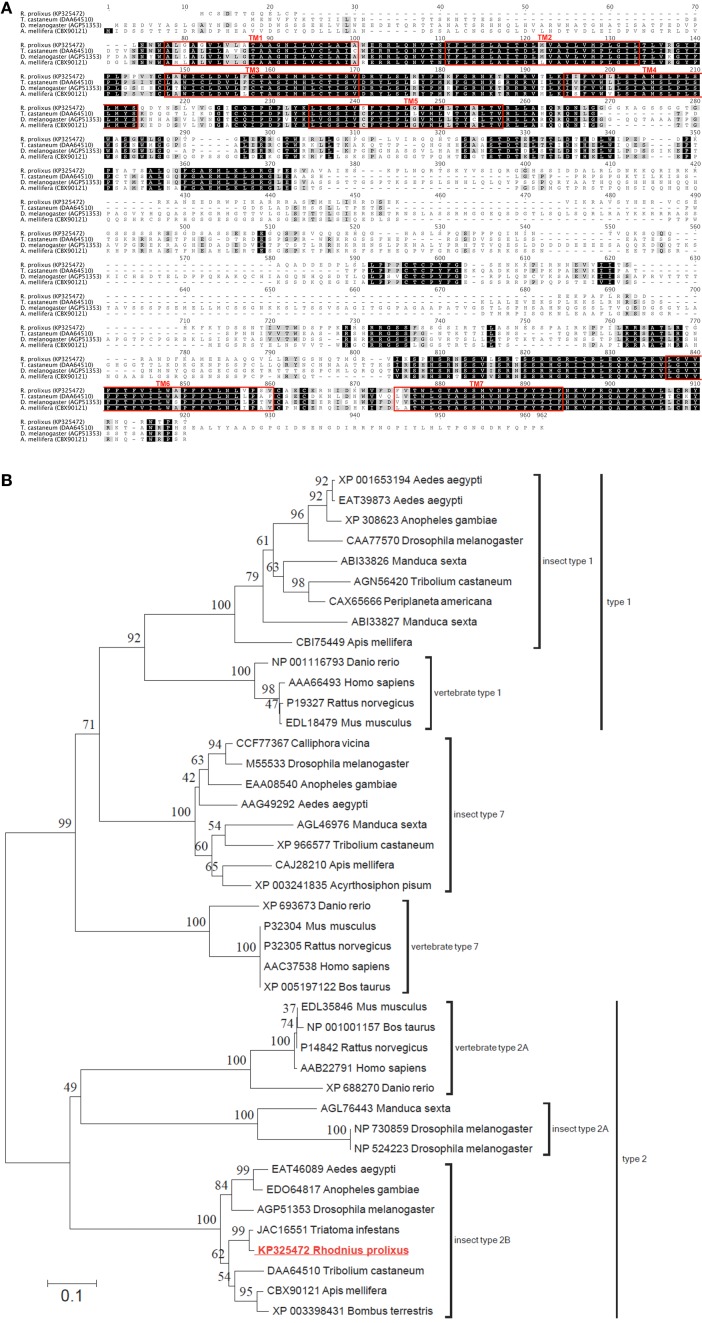
**Protein sequence alignment and phylogenetic analysis of selected serotonin receptors from insects and vertebrates. (A)** ClustalW alignment of selected serotonin type 2b receptors, with regions of predicted transmembrane domains denoted by red outlined boxes. **(B)** Phylogenetic analysis using the neighbor-joining method of the deduced Rhopr5HTR2b receptor protein sequence based on the cloned cDNA. Branch lengths on the tree are representative of the number of average number of amino acid substitutions per site. Percent bootstrap support for the clustering of the related sequences is indicated by the numbers adjacent to the nodes. Each protein sequence included in the analysis is identified by the GenBank accession number and the species name. The *R. prolixus* serotonin receptor identified in the current study (shown in red font) clusters within the clade including insect type 2b serotonin receptors.

Utilizing the ClustalW plugin in Geneious software, the *R. prolixus* 5HTR2b deduced protein sequence was compared to highly similar type 2b receptors from other insects including *Tribolium castaneum* [Genbank accession DAA64510; (Hauser et al., 2008)], *A. mellifera* [Genbank accession CBX90121; (Thamm et al., 2013)] and *D. melanogaster* [Genbank accession AGP51353; (Gasque et al., 2013)]. High sequence similarity or identity was observed largely over the predicted transmembrane domains (Figure 2A). In addition, regions of high similarity and identity were also observed over intracellular and extracellular loops, which may indicate regions essential for intracellular signaling and ligand recognition, respectively. In addition to the potential phosphorylation sites discussed above, the C-terminus contains a conserved cysteine residue (Cys_692_ in Rhopr5HTR2b) that may undergo post-translational palmitoylation (Thamm et al., 2013). The addition of palmitate groups could control multiple receptor functions (Gorinski and Ponimaskin, 2013) including receptor dimerization or G protein association (Zheng et al., 2012) since palmitoylation can create different intracellular loop arrangements with palmitate groups penetrating the lipid bilayer (Goddard and Watts, 2012). Phylogenetic analysis of the *R. prolixus* 5HTR2b receptor deduced protein sequence, which included a comparison to representative sequences from the three main families of insect serotonin receptors and structurally related vertebrate homologs, yielded a tree strongly supporting Rhopr5HTR2b as a member of the insect type-2b receptor subfamily (Figure 2B).

### Transcript expression analyses of *Rhopr5HTR2b*

**Figure 3:**
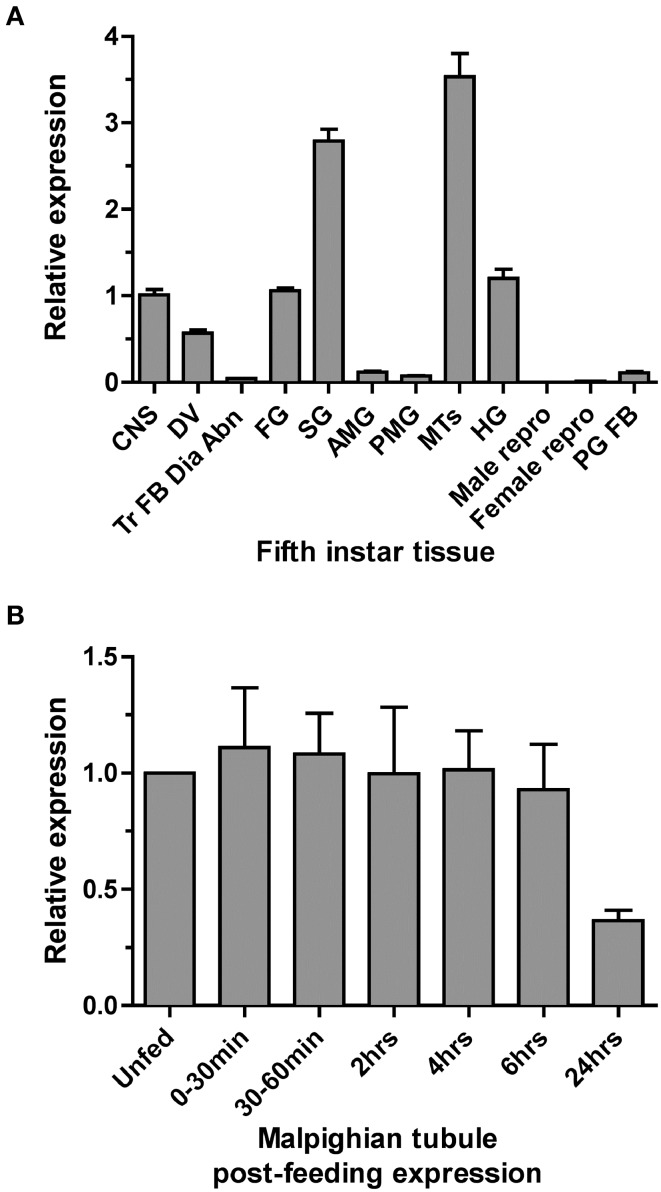
***Rhodnius prolixus* serotonin receptor type 2b (*Rhopr5HTR2b*) transcript expression profile in fifth instar tissues determined by qPCR. (A)** *Rhopr5HTR2b* transcript expression examined in fifth-instar tissues pooled from both male and female insects (except for sex-specific reproductive tissues). Relative expression is shown relative to levels in the CNS. **(B)** *Rhopr5HTR2b* transcript expression in MTs from unfed fifth instar and insects dissected at several time points up to 24 h post-blood meal engorgement. CNS, central nervous system; DV, dorsal vessel; Tr FB Dia Abn, trachea, fat body, dorsal/ventral diaphragm, and abdominal nerves; FG, foregut; SG, salivary gland; AMG, anterior midgut; PMG, posterior midgut; MTs, Malpighian tubule; HG, hindgut; PG FB, prothoracic gland and associated fat body; Male repro, male reproductive tissue; Female repro, female reproductive tissue.

Quantitative PCR (qPCR) was used to examine the expression profile of the *Rhopr5HTR2b* receptor in fifth instar tissues. Highest transcript enrichment is found in the MTs and the salivary glands and lower levels, approximately three-fold less abundance, is found in the CNS, foregut, hindgut and dorsal vessel (Figure 3A). Expression was very low or absent in all other tissues examined which included midgut, reproductive tissues, prothoracic glands (with associated fat body) and a pool of tissue comprised of trachea, fat body, diaphragm, and abdominal nerves.

Considering the highest enrichment observed in MTs and the diuretic role of serotonin in the rapid post-feeding diuresis, we examined the expression profile between unfed and recently fed fifth-instar insects. We observed no changes in *Rhopr5HTR2b* transcript abundance between unfed and time points up to 6 h post-feeding (Figure 3B). Interestingly, expression is somewhat lower at 24 h post-feeding (by approximately 60%), but this was not significantly different from unfed insects (One-Way ANOVA, *p* > 0.05).

### Cell culture-based receptor functional assay

**Figure 4:**
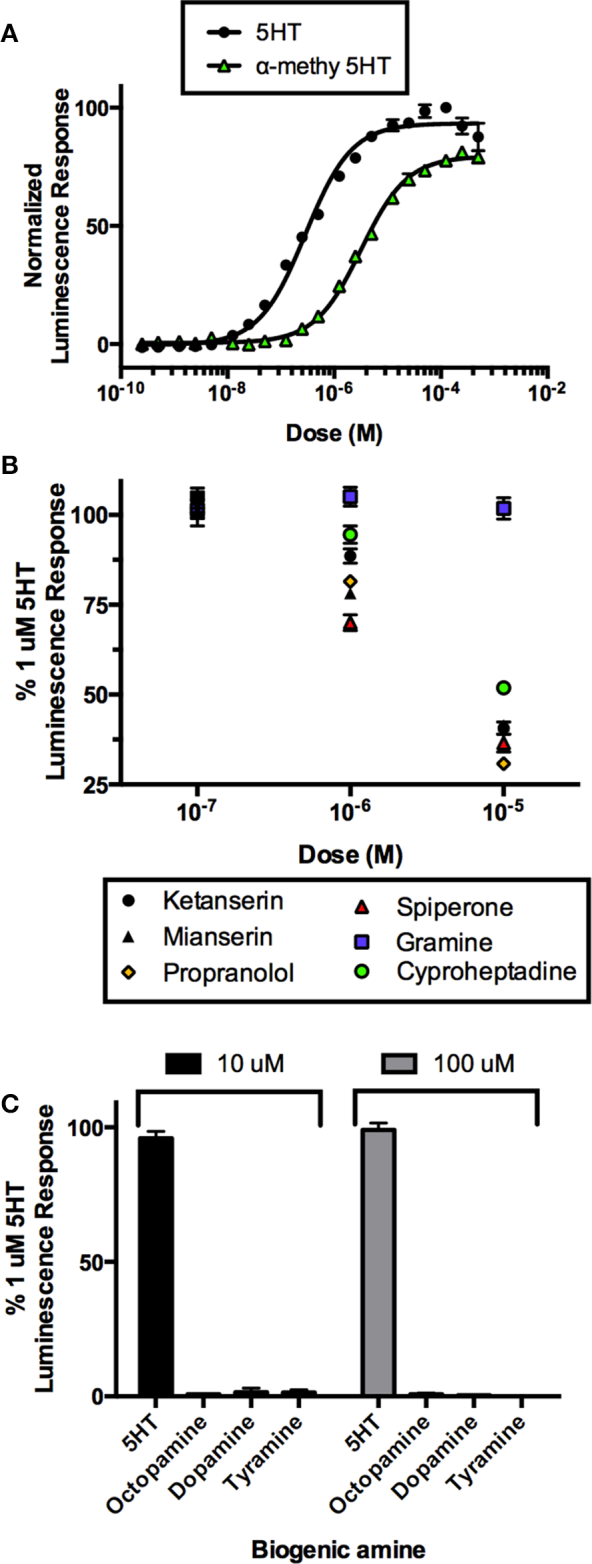
**Heterologous functional assay of the *R. prolixus* serotonin type 2b receptor (Rhopr5HTR2b) in CHOK1-aeq cells. (A)** Dose-response curve demonstrating activity of serotonin and an agonist, alpha-methyl 5-HT, on the expressed Rhopr5HTR2b receptor. **(B)** Several serotonin receptor antagonists blocked the receptor-induced luminescent response, while gramine had no significant effect. **(C)** All other insect biogenic amines are inactive on the expressed Rhopr5HTR2b receptor.

In order to confirm this receptor as a *bona fide* target of serotonergic signaling, we utilized mammalian cell culture as a model to confirm the ligand and pharmacological sensitivity of this putative serotonin receptor. Recombinant CHOK1-aeq cells stably expressing the jellyfish photoprotein apoaequorin (Paluzzi et al., 2012) were used for transient expression of the putative *R. prolixus* serotonin receptor (Rhopr5HT2b) using the pcDNA3.1 mammalian expression vector. CHOK1-aeq cells transiently expressing Rhopr5HTR2b yielded dose-dependent luminescence responses to serotonin (Figure 4A) with threshold activity in the low nanomolar range (EC_50_ = 201 nM). We also evaluated the vertebrate serotonin receptor type-2 agonist, alpha-methyl serotonin, which similarly yielded a dose-dependent luminescence response, albeit with lower potency (EC_50_ = 3.2 μM). Application of serotonin or alpha-methyl serotonin to untransfected cells or cells transfected with empty vector (lacking the *R. prolixus* 5HTR2b cDNA) did not exhibit a luminescence response (data not shown).

In order to more fully characterize and better classify the receptor subtype, we tested a variety of known serotonin receptor antagonists in the presence of a sub-maximal dose of serotonin (1 μM). All drugs were ineffective at a dose 100 nM as luminescence output was not different from controls (Figure 4B). Lower doses were also tested but similarly showed no inhibitory activity (data not shown). Increasing the concentration of the putative antagonists to 1 μM showed differential drug sensitivity. Specifically, no change in luminescence response was seen in the presence of gramine but variable levels of inhibition (30-5% inhibition) were observed with spiperone, mianserin, propranolol, ketanserin, and cyproheptadine. At the highest tested concentration of antagonists (10 μM), gramine still had no effect on the luminescence response induced by serotonin whereas all remaining antagonists caused a greater reduction in the luminescence response (~70–50% inhibition) observed for propranolol, spiperone, ketanserin, mianserin, and cyproheptadine (Figure 4B).

We examined the specificity of the 5HTR2 receptor for its aminergic ligand, 5-HT. Comparing to the luminescence output of 5HTR2b-transfected CHOK1-aeq cells treated with 1 μM 5HT, we tested other biogenic amines (Figure 4C) including dopamine, octopamine and tyramine at two doses (10 and 100 μM) expected to be saturating on their respective natural receptor targets. Our receptor yielded no response to any of the other amines tested with the exception of its confirmed natural ligand, 5-HT.

### Dorsal vessel contraction assay

**Figure 5:**
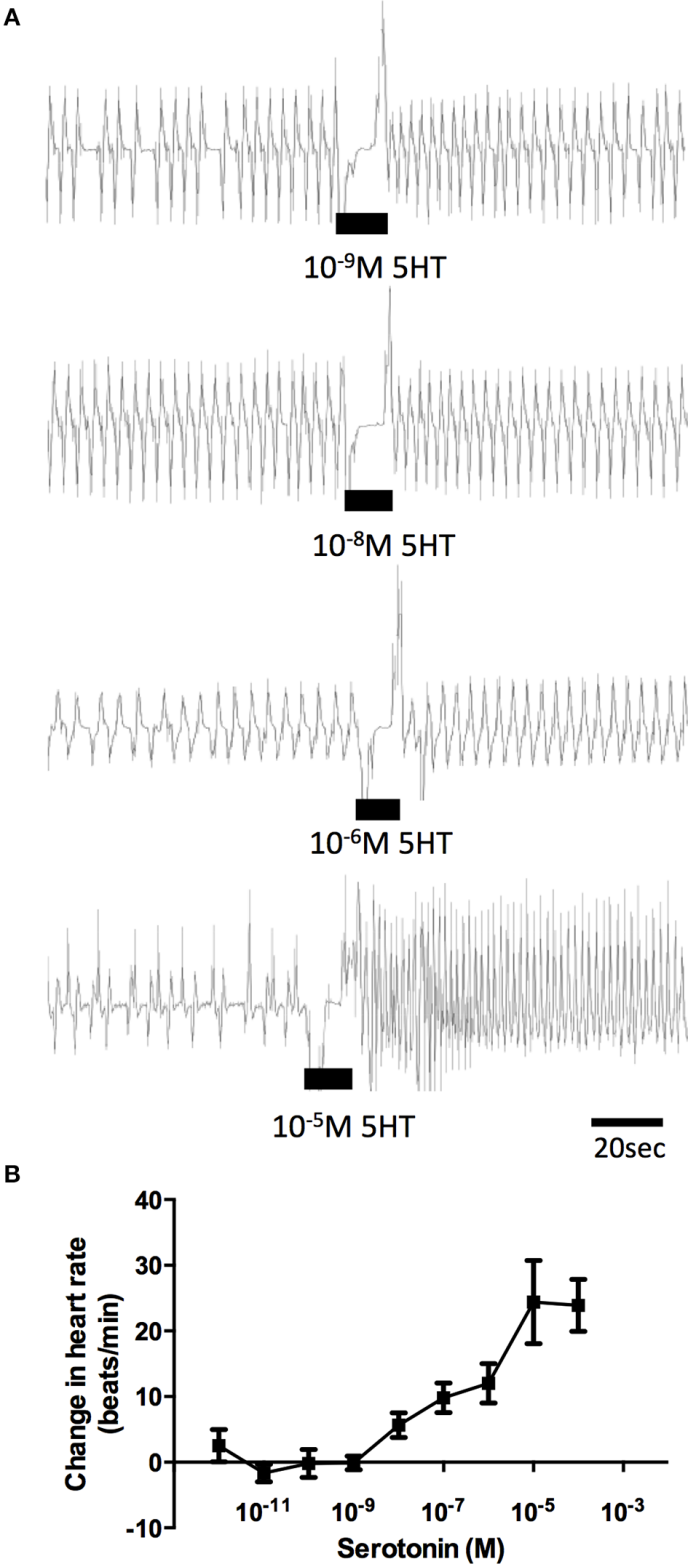
**Dose-dependent activity of serotonin on semi-isolated dorsal vessel preparations *in vitro*. (A)** Sample traces of various concentrations of serotonin, where the black bar denotes time of application of serotonin to a preparation displaying spontaneous heart beats in saline. **(B)** Summary of dose-dependent effects of serotonin on the heart bioassay plotted as change in the heart rate (beats/min) from saline control. Data points are means ± standard error (*n* = 8–12 for each data point).

The effects of serotonin on semi-isolated dorsal vessel preparations confirmed a stimulatory effect on the frequency of heart contractions (Figure 5A). Serotonin dose-dependently increased the rate of dorsal vessel contractions (Figure 5B) with threshold in the low nanomolar range (1–10 nM) and maximal effect observed in the mid micromolar range (10 μM). The average frequency of dorsal vessel contractions under physiological saline alone was 14.96 ± 0.7 beats/min and the most potent dose of serotonin (10 μM) led to an increase in contraction frequency by 24.5 ± 4.4 beats/min, which represents an approximate 165% increase relative to control.

**Figure 6:**
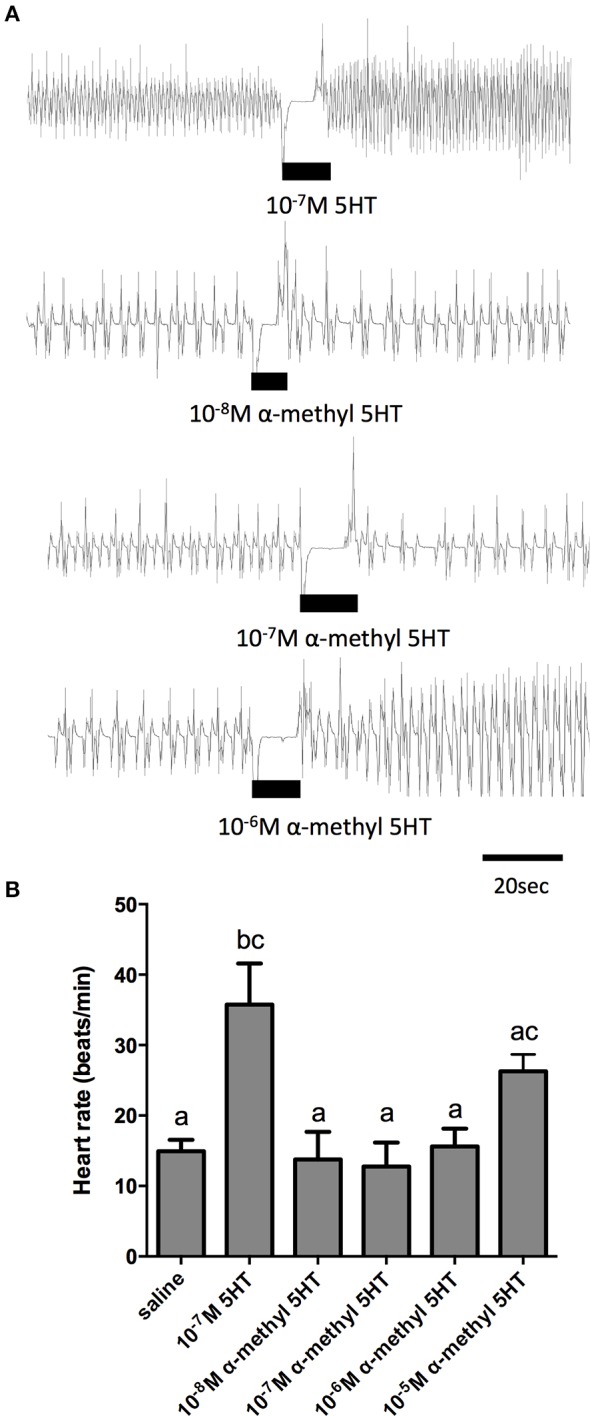
**The serotonin type 2 receptor agonist, α–methyl serotonin, is a weak cardioactive factor in *R. prolixus*. (A)** Sample traces of 100 nM serotonin and selected concentrations of α–methyl serotonin. Black bars denotes application of serotonin or agonist at specified dose to preparations displaying spontaneous heart beats in saline. **(B)** Bar graph summary of the frequency of heart contractions (beats/min) under saline, serotonin or various concentrations of α–methyl serotonin. Columns marked with different letters are significantly different (ANOVA, Tukey-Kramer multiple comparison test, *P* < 0.05). Data points are means ± standard error (*n* = 8 for each data point).

Given the sensitivity of the receptor isolated in this study to alpha-methyl serotonin (a serotonin type-2 receptor agonist) when examined in the cell culture-based receptor functional assay, we also tested this compound on the dorsal vessel bioassay. We selected an intermediate dose of serotonin (100 nM), which revealed a doubling in the frequency of dorsal vessel contractions (Figures 5B, 6A). As can be seen, alpha-methyl serotonin was not an effective agonist on the serotonin receptor type facilitating an increased heart rate (Figure 6A). We did observe some minor increases in the frequency of dorsal vessel contractions (i.e., heart rate) at the highest doses tested (10 μM); however, these were not significantly different from saline control treatments (Figure 6B).

**Figure 7:**
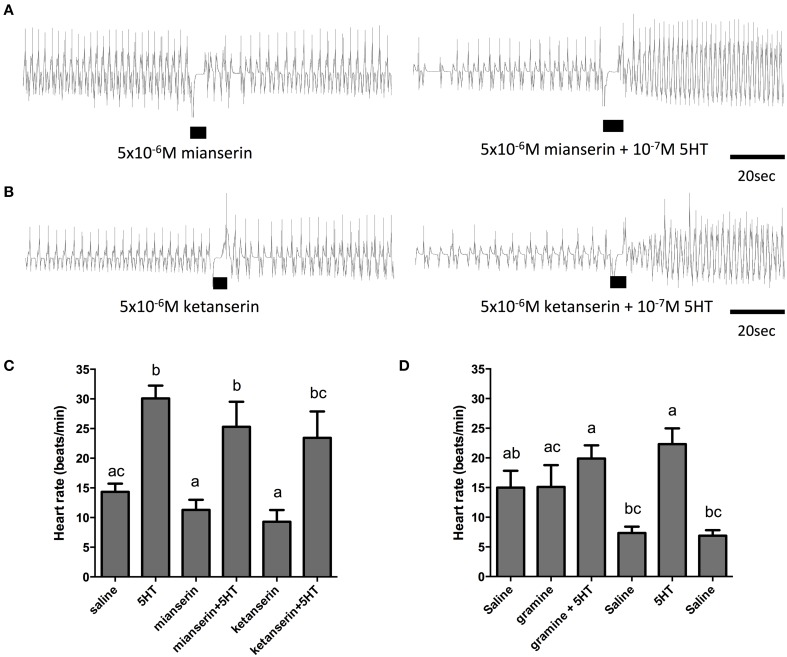
**The serotonin type 2 receptor antagonists, mianserin and ketanserin, are not effective inhibitors of serotonin-stimulated increases in heart rate. (A)** Sample traces of 5 μM mianserin application in isolation and in the presence of 100 nM serotonin. **(B)** Sample traces of 5 μM ketanserin application in isolation and in the presence of 100 nM serotonin. Black bars denotes application of antagonist or antagonist with serotonin at specified concentrations, trace to the left of the black bar represents spontaneous myogenic contractions in saline and to the right of the black bar is the response to serotonin or agonist application. **(C,D)** Bar graph summary of the frequency of heart contractions (beats/min) under saline, serotonin alone, single antagonists alone or in combination with serotonin. Columns marked with different letters are significantly different (ANOVA, Tukey-Kramer multiple comparison test, *P* < 0.05). Data points are means ± standard error (*n* = 6 for each data point for assessments with mianserin and ketanserin; *n* = 9 for each data point for assessments with gramine).

In order to further elucidate the receptor type mediating the cardioacceleratory action in *R. prolixus*, we tested a subset of the receptor antagonists utilized in the cell culture-based receptor functional assay, namely mianserin, ketanserin and gramine. As shown in the sample traces and the summarizing bar graph, application of mianserin and ketanserin alone at a dose of 5 μM did not significantly affect the heart rate (Figures 7A–C). As shown earlier, 100 nM serotonin significantly increased the heart rate (Figure 7C) and also significantly increased the heart rate in the presence of the candidate antagonists mianserin and ketanserin (Figures 7A–C). Thus, unlike the result observed in the receptor functional assay, these two candidate antagonists were not effective at blocking serotonin's cardioacceleratory activity. We also examined the effects of gramine, which was not an effective antagonist on the isolated receptor when assessed using the cell culture-based functional assay (Figure 4C). When tested on its own, 50 μM gramine had no effect on heart rate. In the presence of 100 nM serotonin and 50 μM gramine, only a small increase to heart rate was observed (Figure 7D), which was not significantly different from the saline control. Upon wash off with saline, heart rate decreased and subsequent application of 100 nM serotonin alone led to an increased frequency of dorsal vessel contractions (i.e., elevated heart rate). Unlike our observations of the isolated receptor in the receptor functional assay, gramine appears to be a weak antagonist of the receptor controlling serotonin's chronotropic activity on the dorsal vessel.

## Discussion

Diuresis in *R. prolixus* is under the control of at least two diuretic hormones, which includes the biogenic amine serotonin and RhoprCRF/DH (Lange et al., 1989; Maddrell et al., 1991; Te Brugge et al., 2011). In addition to its established role in stimulating fluid secretion by upper MTs, serotonin has a number of additional effects on a variety of tissues in *R. prolixus* (Orchard, 2006, 2009) as well as in other insects (Molaei and Lange, 2003; Dacks et al., 2006a; Wang et al., 2013; French et al., 2014; Majeed et al., 2014) that are mediated through a neurotransmitter, neuromodulator or neurohormone mechanism. We have identified a serotonin receptor that shares sequence characteristics most consistent with the insect type 2b serotonin receptors described previously (Hauser et al., 2008; Gasque et al., 2013; Thamm et al., 2013). The genomic organization is similar to that described in other insects, having either six (Thamm et al., 2013) or seven exons (Hauser et al., 2008; Gasque et al., 2013); however, the transmembrane domains in the *R. prolixus* 5HTR2b gene have different exon localizations (for comparison see Hauser et al., 2006; Thamm et al., 2013). Phylogenetic analysis also supports the notion that the receptor isolated here belongs to the insect serotonin receptor type-2b subfamily—a nomenclature used to differentiate members of this subfamily from the insect serotonin receptor type-2a subfamily (Colas et al., 1995; Gasque et al., 2013).

Transcript expression profiling of *Rhopr5HTR2b* in various tissues of fifth instar stage *R. prolixus* revealed greatest enrichment in MTs and salivary glands. Pharmacological analyses in the heterologous system suggests this receptor could be the target of serotonin acting as a diuretic hormone enabling increased solute and water transport by the MTs following a blood meal. This notion is supported by previous studies that found ketanserin and spiperone are potent antagonists of serotonin-stimulated fluid secretion by MTs (Maddrell et al., 1991; Te Brugge et al., 2001), which were also the most active antagonists on the receptor we identified in this study. Interestingly, relative to the unfed animal, it appears there is no change in receptor transcript abundance over the first 24 h after blood meal engorgement, suggesting the Malpighian tubule serotonin receptor that responds to haemolymph-borne serotonin is available in advance of feeding to ensure a prompt response to initiate the rapid post-feeding diuresis.

The pharmacological characteristics of the serotonin receptor type on *R. prolixus* salivary glands are not known. In *Calliphora vicina* salivary glands, different serotonin receptor types may regulate salivation since ketanserin is not effective at blocking serotonin-stimulated fluid secretion by isolated salivary glands (Maddrell et al., 1991), although serotonin-stimulated salivation in *C. vicina* is significantly inhibited by gramine (Berridge, 1972; Trimmer, 1985). Gramine was not effective at inhibiting the activation of the Rhopr5HTR2b receptor functionally analyzed here. Two receptors have been recently identified in blowfly salivary glands belonging to the serotonin receptor type-2a and type-7 classes (Roser et al., 2012), although both receptors displayed sensitivity to ketanserin (Roser et al., 2012). A different receptor subtype could be expressed in the *R. prolixus* salivary glands where serotonin is delivered in the nerve supply and induces a dose-dependent increase in the frequency and amplitude of phasic muscle contractions (Orchard and Te Brugge, 2002). Serotonin has also been reported to dose-dependently stimulate secretion of saliva (Orchard, 2006).

*Rhopr5HTR2b* transcript expression was also detected in the CNS, dorsal vessel, foregut and hindgut of fifth instar *R. prolixus*. In the nervous system of insects, serotonin is known to play a variety of roles that includes regulation of feeding (Falibene et al., 2012; French et al., 2014), motor output and locomotion (Claassen and Kammer, 1986; Silva et al., 2014), control of clock neurons (Hamasaka and Nassel, 2006; Kolodziejczyk and Nassel, 2011), and olfactory function (Mercer and Menzel, 1982; Kloppenburg et al., 1999; Dacks et al., 2006a; Tsuji et al., 2007; Kloppenburg and Mercer, 2008; Siju et al., 2008; Zhao and Berg, 2009; Watanabe et al., 2014). In *R. prolixus*, serotonin's distribution in the CNS is extensive, with approximately 150 neurons identified, whose projections and arborizations suggest that this neurochemical has a multitude of central as well as peripheral roles (Lange et al., 1988). A subset of the peripheral serotonin-like immunoreactive projections in *R. prolixus* are associated with regions of the gut including the foregut and hindgut (Orchard et al., 1988; Orchard, 2006), These regions of the gut also demonstrated *Rhopr5HTR2b* transcript enrichment, suggesting serotonin's roles could be mediated by this receptor in these tissues. In contrast, serotonin's action on the anterior midgut as a regulator of transepithelial transport (Farmer et al., 1981) and muscle contraction (Te Brugge et al., 2009) may not involve the identified receptor since no transcript was detected in this tissue. It is possible, however, that a closely related receptor (e.g., type 2a receptor class) may control these serotonergic actions in the anterior midgut since cAMP responsiveness to serotonin showed a similar pharmacological profile, including sensitivity to the agonist α-methyl serotonin and inhibition by antagonists including ketanserin, mianserin and cyproheptadine, while the serotonin-induced increase of cAMP in the anterior midgut was not affected by spiperone (Barrett et al., 1993), one of the more active antagonists effective on Rhopr5HTR2b. Spiperone may be useful in discriminating between these closely related receptor subtypes.

The dorsal vessel is the primary circulatory organ in insects and is regulated by a variety of cardioactive factors, such as serotonin (Chiang et al., 1992; Zornik et al., 1999; Koladich et al., 2002; Dasari and Cooper, 2006; Feliciano et al., 2011). As previously reported (Chiang et al., 1992; Orchard, 2006), serotonin stimulates increases in heart rate in the low nanomolar range (threshold between 1 and 10 nM). In contrast to the pharmacological sensitivity of Rhopr5HTR2b as determined through the heterologous functional assay, the cardioacceleratory activity of serotonin had a unique pharmacological profile. High doses of the type 2 agonist, alpha-methyl serotonin, were not effective in significantly modifying the frequency of heart contractions. In addition, the dorsal vessel bioassay revealed insensitivity to ketanserin and mianserin. Taken together, these results suggest that at least two distinct serotonin receptors control critical physiological functions in this blood-feeding insect, one of which is Rhopr5HTR2b, involved in fluid secretion by the MTs. Other serotonin receptors appear to be involved in absorption by the anterior midgut and cardioacceleratory effects on the dorsal vessel.

### Conflict of interest statement

The authors declare that the research was conducted in the absence of any commercial or financial relationships that could be construed as a potential conflict of interest.
